# Design, Synthesis, Molecular Modeling, *In Silico* ADME Studies and Anti-HIV-1 Assay of New Diazocoumarin Derivatives

**Published:** 2018

**Authors:** Zeynab Alimi Livani, Mahdieh Safakish, Zahra Hajimahdi, Sepehr Soleymani, Rezvan Zabihollahi, Mohammad Reza Aghasadeghi, Eskandanr Alipour, Afshin Zarghi

**Affiliations:** a *Department of Organic Chemistry, Tehran North Branch, Islamic Azad University, Tehran, Iran.*; b *Department of Pharmaceutical Chemistry, School of Pharmacy, Shahid Beheshti University of Medical Sciences, Tehran, Iran.*; c *Hepatitis and AIDS department, Pasteur institute of Iran, Tehran, Iran.*

**Keywords:** Anti-HIV, Diazocoumarin, 1, 3, 4-Oxadiazole, Synthesis, Docking, ADME

## Abstract

Some new diazo incorporated coumarin compounds were designed and synthesized to evaluate their anti-HIV activity. Overall, compounds were active against HIV at 100 μM. Additionally, no cytotoxic effect was observed at this concentration. The compound with 4-chlorobenzyl group indicated the best anti-HIV activity (52%). Docking studies using the later crystallographic data available for PFV integrase showed similar binding modes to HIV-1 integrase inhibitors. On the basis of these data, nitrogen atoms of 1,3,4-oxadiazole ring have been involved in the Mg^2+^ chelation and 4-chlorobenzyl group occupies the same position as 4-flourobenzyl group of raltegravir in the active site. In addition, *in silico* ADME assay demonstrated favorable physicochemical properties for the new designed compounds. Thus, synthesized structures could be introduced as a novel template for designing safe anti-HIV compounds with integrase inhibitory potential.

## Introduction

**Figure 1:**
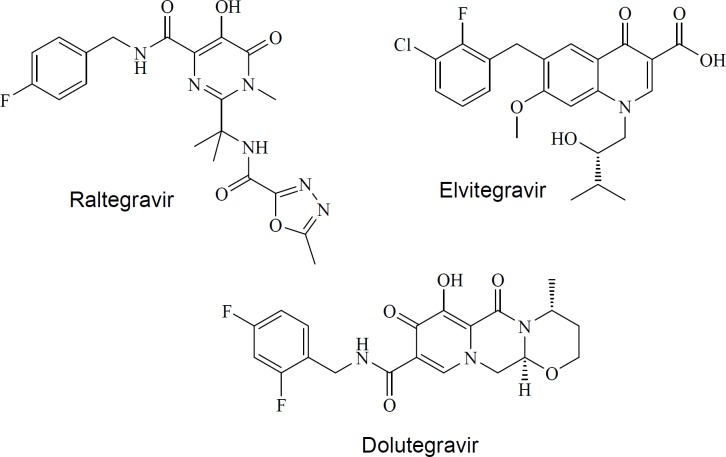
FDA-approved INSTIs

Acquired Immunodeficiency Syndrome (AIDS) is the second cause of death from infectious diseases in low income countries (1). Human Immunodeficiency Virus (HIV) is recognized as the infective agent. Current anti-HIV drugs target different stages of HIV life cycle including absorption, fusion, reverse transcription, integration, and proteolytic cleavage (2-4). Although there is a plethora of anti-HIV drugs, drug toxicity, viral resistance, and patient incompliance has been limited anti-HIV therapy (5). Altogether, there is a demand for novel drugs from alternative scaffolds. HIV-1 integrase is an essential enzyme which catalyzes the integration of viral DNA into the host cellular DNA. This process is separated into 2 steps. First, 3′-processing resulting in the creation of free hydroxyl groups at reverse-transcribed proviral cDNA 3′-ends. During the second step (strand transfer), integrase catalyzes the insertion of the 3′-processed viral DNA into the host cell genome (6, 7). FDA-approved integrase inhibitors (raltegravir (8, 9), elvitegravir (10, 11) and dolutegravir (12, 13) in Figure 1) compete with host DNA in the IN active site, thus specifically inhibiting the strand transfer step (14). Development of integrase strand transfer inhibitors (INSTIs) represents an attractive research area in the field of HIV-1 drug discovery because there is no host cell equivalent for HIV-1 integrase enzyme (15).

The integrase strand transfer inhibitors (INSTIs) bind in the interface of the viral DNA and catalytic DDE motif. For this reason, they could be classified as interfacial inhibitors. The DDE motif contains a triad of three conserved acidic residues (Asp-64, Asp-116, and Glu-152) coordinating 2 Mg^2+^ cofactors which are critical for the catalytic activity (16). There are various chemical classes of INSTIs such as oligonucleotides, peptides, hydroxylated aromatics, hydrazides, quinoline, and quinolones. All specific INSTIs feature a metal chelating motif that complexes 2 Mg^2+ ^cofactors and a hydrophobic terminal benzyl moiety (17-19). 

**Figure 2:**
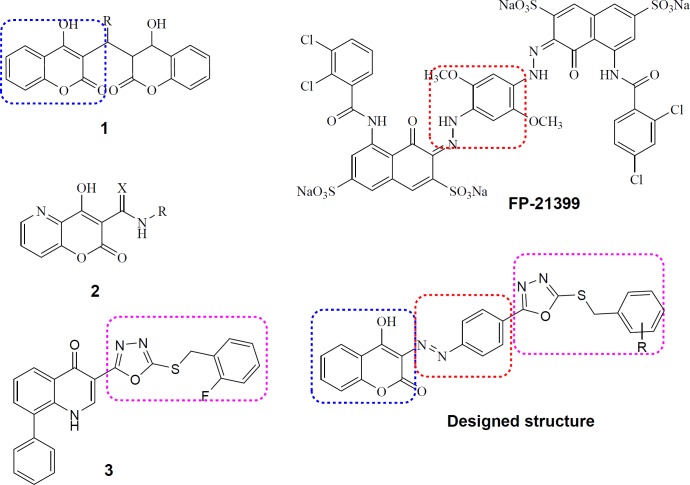
Lead anti-HIV compounds (1, 2, 3 and FP-21399) and our designed structure

Coumarin is a well-known drug scaffold which displays versatile biological activities especially substantial anti-HIV activity (20). Notably, IN inhibitory activity has been reported for coumarin dimmers (**1**, Figure 2) (21) and 4-hydroxy-5-azacoumarin-3-carbox(thio)-amides (**2**, Figure 2) (22). Furthermore, the anti-HIV activity of a bis-azo compound (FP-21399, Figure 2) has been evidenced (23). With regard to these data we selected coumarin scaffold as a core moiety to design new anti-HIV compounds. The decision for selecting substitutions on coumarin ring was carried out from the information obtained through our previous studies. Recently, we reported development of 8-phenylquinolin-4(1*H*)-one derivatives possessing 1,3,4-oxadiazole ring as metal-chelating moiety along with a benzyl part necessary for HIV-1 IN inhibitory activity. Among them, compound **3** (Figure 2) displayed promising anti-HIV-1 activity with EC_50_ = 50 µM and no considerable cytotoxicity (CC_50_ > 100 µM) (24). SAR analysis showed that the incorporation of electronegative substitutions like halogens (-F, -Cl) at proper positions of benzyl ring would improve inhibitory activity (25). In this study, we merged coumarin core with a 5-(halo-substituted) benzylthio-1,3,4-oxadiazole motif through a diazo-phenyl linker (Designed structure, Figure 2) to present new structure for anti-HIV activity. Some compounds containing alkyl substitution such as methyl, ethyl, and propyl instead of benzyl ring at C-5 position of 1,3,4-oxadiazol ring were also designed. Using wide range of substitutions would help us to perform a SAR analysis on this series. 

The overall shape and functionalities of designed structure particularly resemble those of compounds **1** and **3**, suggesting that our novel designed compounds possess ability to be promising anti-HIV agents.

## Experimental

*Chemistry*

**Scheme 1:**
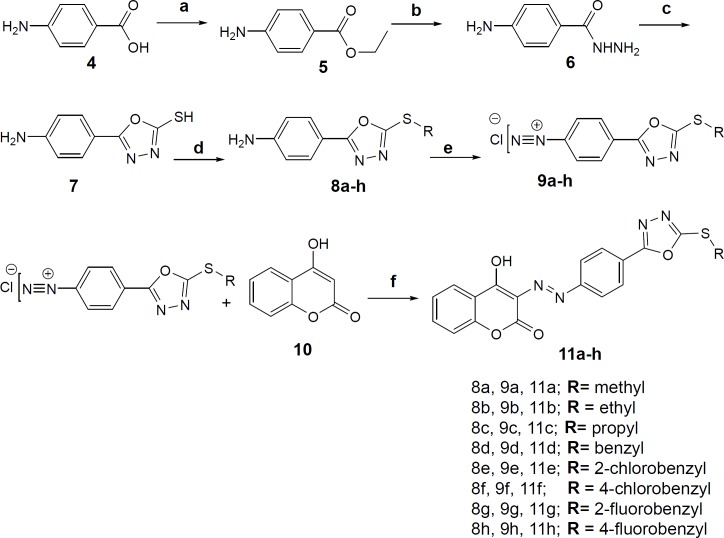
**Reagents and conditions: (**a**) ethanol, sulfuric acid, reflux, 24h (**b**) ethanol, NH**_2_**-NH**_2_**.OH (6 eq.), reflux, 96h (**c**) ethanol, CS**_2_**, KOH, reflux, 4h (**d**) methanol, 10% aq. NaOH, substituted alkyl/aryl halides, r.t (**e**) **1. **6M hydrochloric acid **2.** 10% sodium nitrite, 0-5 °C (**f**) 10% aq. Na**_2_**CO**_3_**, stir, 0-5 °C**

3-((4-(5-(substitutedaryl/alkyl)-1,3,4-oxadiazol-2-yl) phenyl) diazenyl)-4-hydroxy-2*H*-chromen-2-onederivatives were synthesized according to the scheme 1. First, Ethyl 4-aminobenzoate was prepared from 4-aminobenzoic acid (**4**) and ethanol in a routine Fischer esterification reaction under sulfuric acid catalysis. Ethyl 4-aminobenzoate (**5**) was converted to 4-aminobenzohydrazide (**6**) by refluxing in absolute ethanol and excess of hydrazine hydrate. The 2-mercaptho-1,3,4-oxadiazole ring (7) was closed on the carbohydrazide intermediate in the presence of carbon disulfide and alcoholic potash according to procedure described in references (26, 27). S-alkylation/benzylation reaction was performed with alkyl/ substituted benzyl halides in methanol as the solvent and 10% sodium hydroxide solution to afford derivatives **8a-h**. The moderate basicity of the reaction medium is essential to gain the mono S-alkyl/benzyl product rather than both S- and N-alkyl/benzyl substituted product. The diazonium salts of compounds (**9a-h**) were prepared in 6 M hydrochloric acid and 10% sodium nitrite aqueous solution at 0-5 °C. The final derivatives **11a-h** were obtained through coupling reaction of the diazonium salts with 4-hydroxycoumarin (**10**) in aqueous sodium carbonate solution at neutral pH.

*Materials*

All reagents purchased from the Aldrich (USA) or Merck (Germany) Chemical Company and were used without further purifications.

*General*

Melting points (mp) were determined using a Thomas Hoover capillary apparatus (Philadelphia, USA). Infrared spectra were acquired on a Perkin-Elmer 1420 ratio recording spectrometer. A Bruker FT-500 MHz instrument (Brucker Biosciences, USA) was used to acquire ^1^HNMR; Chloroform-D used as solvent. Coupling constant (*J*) values are estimated in hertz (Hz) and spin multiples are given as brs (broad singlet), s (singlet), d (double), t (triplet), q (quartet), m (multiplet), and br (broad). The mass spectral measurements were performed on an 6410 Agilent LCMS triple quadrupole mass spectrometer (LCMS) with an electrospray ionization (ESI) interface.

*Ethyl 4-aminobenzoate (* ***5*** *)*

Yield 74%; White crystalline powder; mp 89.2-91.0 °C; IR (KBr disk): υ (cm^-1^) 3424- 3348 (NH_2_), 1682 (C=O); LC-MS (ESI) *m/z*: 166.1 (M+1, 100). 

*4-Aminobenzohydrazide (* ***6*** *)*

Yield 82%; White crystalline powder; mp 216.8-218.3 °C; IR (KBr disk): υ (cm^-1^) 3432- 3350 (NH_2_), 3307-3278 (-CO-NH*NH*_2_), 3239 (-CO-*NH*NH_2_), 1603 (C=O); LC-MS (ESI) *m/z*: 152.0 (M+1, 100). 

*5-(4-Aminophenyl)-1,3,4-oxadiazole-2-thiol (* ***7*** *)*

To a solution of compound **6** (6 mmol) and potassium hydroxide (1.23 g) in absolute ethanol (50 mL), carbon disulfide (8.2 mL) was added in an ice bath. The mixture was refluxed at 50-60 °C for 4 hours. Then, it was cooled to room temperature and the solvent was evaporated to dryness. The crude suspended in 7 mL water and acidified with 3 M hydrochloric acid until pH 4.5. The yellow precipitate was filtered and washed with water. Yield 73%; Yellow powder; mp 197-199 °C; IR (KBr disk): υ (cm^-1^) 3356- 3440 (NH_2_); LC-MS (ESI) *m/z*: 194.0 (M+1, 100).

*General procedure for preparation of 4-(5-(substituted thio)-1,3,4-oxadiazol-2-yl) aniline (* ***8a-h*** *)*

To a solution of compound **7** (1.55 mmol) in methanol (4 mL) sodium hydroxide (10%, 0.12 mL) was added. The mixture was cooled in an ice bath and substituted benzyl/alkyl halide (1.55 mmol) was added dropwise and stirred at room temperature. After completion of the reaction, the precipitate was filtered, rinsed with water, and crystallized from ethanol.

*4-(5-(Methylthio)-1,3,4-oxadiazol-2-yl)aniline (* ***8a*** *)*

Yield 87%; Yellow crystalline powder; mp 105.8-107.6 °C; IR (KBr disk): υ (cm^-1^) 3327- 3425 (NH_2_); LC-MS (ESI) *m/z*: 207.9 (M+1, 100).

*4-(5-(Ethylthio)-1,3,4-oxadiazol-2-yl)aniline (* ***8b*** *)*

Yield 73%; Yellow crystalline powder; mp 80.4-83.2 °C; IR (KBr disk): υ (cm^-1^) 3374-3444 (NH_2_); LC-MS (ESI) *m/z*: 221.9 (M+1, 100). 

*4-(5-(Propylthio)-1,3,4-oxadiazol-2-yl)aniline (* ***8c*** *)*

Yield 67%; Yellow crystalline powder; mp 105.1-106.8 °C; IR (KBr disk): υ (cm^-1^) 3333-3478 (NH_2_); LC-MS (ESI) *m/z*: 236.9 (M+1, 100).

*4-(5-(Benzylthio)-1,3,4-oxadiazol-2-yl)aniline (* ***8d*** *)*

Yield 61%; Yellow crystalline powder; mp 120.8-124 °C; IR (KBr disk): υ (cm^-1^) 3359-3493 (NH_2_); LC-MS (ESI) *m/z*: 284.9 (M+1, 100).

*4-(5-(2-Chlorobenzylthio)-1,3,4-oxadiazol-2-yl)aniline (* ***8e*** *)*

Yield 59%; Yellow crystalline powder; mp 117.8-118.9 °C; IR (KBr disk): υ (cm^-1^) 3337-3399 (NH_2_); LC-MS (ESI) *m/z*: 318.9 (M+1, 100).

*4-(5-(4-Chlorobenzylthio)-1,3,4-oxadiazol-2-yl) aniline (* ***8f*** *)*

Yield 65%; Yellow crystalline powder; mp 141.8-144.3 °C; IR (KBr disk): υ (cm^-1^) 3349-3417 (NH_2_); LC-MS (ESI) *m/z*: 318.9 (M+1, 100).

*4-(5-(2-Fluorobenzylthio)-1, 3, 4-oxadiazol-2-yl) aniline (* ***8g*** *)*

Yield 55%; Yellow crystalline powder; mp 121.8-123.8 °C; IR (KBr disk): υ (cm^-1^) 3373-3506 (NH_2_); LC-MS (ESI) *m/z*: 302.9 (M+1, 100).

*4-(5-(4-Fluorobenzylthio)-1, 3, 4-oxadiazol-2-yl) aniline (* ***8h*** *)*

Yield 59%; Yellow crystalline powder; mp 104-106 °C; IR (KBr disk): υ (cm^-1^) 3353-3422 (NH_2_); LC-MS (ESI) *m/z*: 302.9 (M+1, 100).

*General procedure for the preparation of (E)-4-hydroxy-3-((4-(5-(substitutedthio)-1, 3, 4-oxadiazol-2-yl) phenyl)diazenyl)-2H-chromen-2-one (* ***11a-h*** *)*

To a cooled solution of substituted compounds **8a-h** (1 mmol) in 6 M hydrochloric acid (2.7 mL), 0.7 mL cooled solution of 10% sodium nitrite was added dropwise in order to keep the temperature less than 5 °C. Then, the mixture was poured into a solution of 4-hydroxy coumarin (**10**) (1 mmol) and sodium carbonate (0.05 g) in water (1.85 mL). The pH of the mixture was adjusted to 7 by addition of sodium acetate. The reaction mixture was stirred in an ice bath. After completion of the reaction, the precipitate was filtered, washed with water and crystallized from acetonitrile. 

*4-Hydroxy-3-((4-(5-(methylthio)-1, 3, 4-oxadiazol-2-yl) phenyl)diazenyl)-2H-chromen-2-one (* ***11a*** *)*

Yield 81%; Orange powder; mp 211.6-212.1 °C; IR (KBr disk): υ (cm^-1^) 1666-1744 (C=O); ^1^HNMR (CDCl_3_, 500 MHz): δ (ppm) 2.59 (3H, s, -CH_3_); 7.32 (1H, d, *J*= 7.5 Hz, H_8_) 7.34-7.39 (1H, m, H_6_), 7.68-7.74 (3H, m, H_7_ & phenyl H_2_ & H_6_), 7.98 (2H, brs, phenyl H_3_ & H_5_), 8.09 (1H, d, *J*= 7.4 Hz, H_5_); LC-MS (ESI) *m/z*: 381.2 (M+1, 100). Anal. Calcd. for C_18_H_12_N_4_O_4_S; C, 56.84; H, 3.18; N, 14.73. Found: C, 56.99; H, 3.32; N, 14.52.

*3-((4-(5-(Ethylthio)-1, 3, 4-oxadiazol-2-yl) phenyl)diazenyl)-4-hydroxy-2H-chromen-2-one (* ***11b*** *)*

Yield 78%; Orange powder; mp 290-291 °C (decomposed); IR (KBr disk): υ (cm^-1^) 1760 (C=O); ^1^HNMR (CDCl_3_, 500 MHz): δ (ppm) 1.53 (3H, t, *J*= 7.3 Hz, -CH_3_), 3.34 (2H, q, *J*= 7.3 Hz, -CH_2_), 7.30 (1H, d, *J*= 8.2 Hz, H_8_), 7.34 (1H, t, *J*= 7.5 Hz, H_6_), 7.70 (1H, t, *J*= 7.3 Hz, H_7_), 7.78 (2H, d, *J*= 8.5 Hz, phenyl H_2_ & H_6_), 8.09-8.14 (3H, m, H_5 _& phenyl H_3_ & H_5_); LC-MS (ESI) *m/z*: 395.0 (M+1, 100). Anal. Calcd. for C_19_H_14_N_4_O_4_S; C, 57.86; H, 3.58; N, 14.21. Found: C, 57.66; H, 3.41; N, 

14.01. 

*4-Hydroxy-3-((4-(5-(propylthio)-1, 3, 4-oxadiazol-2-yl) phenyl)diazenyl)-2H-chromen-2-one (* ***11c*** *)*

Yield 71%; Orange powder; mp 205.5-206.8 °C; IR (KBr disk): υ (cm^-1^) 1732 (C=O); ^1^HNMR (CDCl_3_, 500 MHz): δ (ppm) 1.09 (3H, t, *J*= 7.3 Hz, -CH_3_), 1.89 (2H, sixtet, *J*= 7.3 Hz, CH_3_-C*H*_2_), 3.30 (2H, t, *J*= 7.2 Hz, CH_2_-C*H*_2_), 7.30 (1H, d, *J*= 8.3 Hz, H_8_), 7.34 (1H, t, *J*= 7.5 Hz, H_6_), 7.70 (1H, t, *J*= 8.4 Hz, 7.2, H_7_), 7.78 (2H, d, *J*= 8.6 Hz, phenyl H_2_ & H_6_), 8.09-8.1 (3H, m, H_8 _& phenyl H_3_ & H_5_); LC-MS (ESI) *m/z*: 409.2 (M+1, 100). Anal. Calcd. for C_20_H_16_N_4_O_4_S; C, 58.82; H, 3.95; N, 13.72. Found: 58.61; H, 3.77; N, 13.55.

*3-((4-(5-(Benzylthio)-1, 3, 4-oxadiazol-2-yl) phenyl)diazenyl)-4-hydroxy-2H-chromen-2-one (* ***11d*** *)*

Yield 75%; Orange powder; mp 243.1-243.8 °C; IR (KBr disk): υ (cm^-1^) 1737 (C=O); ^1^HNMR (CDCl_3_, 500 MHz): δ (ppm) 4.58 (2H, s, -CH_2_), 7.35-7.39 (5H, m, H_6_ & H_8_ & benzyl H_3_ & H_4_ & H_5_), 7.50 (2H, d, *J*= 7.2 Hz, benzyl H_2 _& H_6_), 7.74 (1H, m, H_7_), 7.82 (2H, d, *J*= 8.2 Hz, phenyl H_2_ & H_6_), 8.13-8.15 (3H, m, H_5_ & phenyl H_3_ & H_5_); LC-MS (ESI) *m/z*: 284.1 (M+1, 100). Anal. Calcd. for C_24_H_16_N_4_O_4_S; C, 63.15; H, 3.53; N, 12.27. Found: C, 62.95; H, 3.41; N, 12.57.

*3-((4-(5-(2-Chlorobenzylthio)-1, 3, 4-oxadiazol-2-yl) phenyl)diazenyl)-4-hydroxy-2H-chromen-2-one (* ***11e*** *)*

Yield 71%; Orange powder; mp 236.8-237.5 °C; IR (KBr disk): υ (cm^-1^) 1755 (C=O); ^1^HNMR (CDCl_3_, 500 MHz): δ (ppm) 4.69 (2H, s, -CH_2_), 7.30-7.45 (5H, m, H_6_ & H_8 _& 2-Cl-benzyl H_4 _& H_5 _& H_6_), 7.67-7.73 (2H, m, H_7 _& 2-Cl-benzyl H_3_), 7.82 (2H, m, phenyl H_2_ & H_6_), 8.13-8.4 (3H, m, H_5_ & phenyl H_3_ & H_5_), 16.26 (1H, s, -OH); LC-MS (ESI) *m/z*: 491.2 (M+1, 100). Anal. Calcd. for C_24_H_15_ClN_4_O_4_S; C, 58.72; H, 3.08; N, 11.41. Found: C, 58.62; H, 3.28; N, 

11.25. 

*3-((4-(5-(4-Chlorobenzylthio)-1, 3, 4-oxadiazol-2-yl) phenyl)diazenyl)-4-hydroxy-2H-chromen-2-one (* ***11f*** *)*

Yield 74%; Orange powder; mp 260.8-263.8 °C; IR (KBr disk): υ (cm^-1^) 1748 (C=O); ^1^HNMR (CDCl_3_, 500 MHz): δ (ppm) 4.49 (2H, s, -CH_2_), 7.30-7.36 (4H, m, H_6_ & H_8 _& 4-Cl-benzyl H_2_ & H_6_), 7.41 (2H, d, *J*= 8.5, 4-Cl-benzyl H_3 _& H_5_), 7.70 (1H, m, H_7_), 7.78 (2H, d, *J*= 10, phenyl H_2_ & H_6_), 8.09 (3H, m, H_5_ & phenyl H_3_ & H_5_); LC-MS (ESI) *m/z*: 514.3 (M+23, 100). Anal. Calcd. for C_24_H_15_ClN_4_O_4_S; C, 58.72; H, 3.08; N, 11.41. Found: C, 58.98; H, 3.23; N, 11.30.

*3-((4-(5-(2-Fluorobenzylthio)-1, 3, 4-oxadiazol-2-yl) phenyl)diazenyl)-4-hydroxy-2H-chromen-2-one (* ***11g*** *)*

Yield 58%; Orange powder; mp 240.1-242.2 °C; IR (KBr disk): υ (cm^-1^) 1752 (C=O); ^1^HNMR (CDCl_3_, 500 MHz): δ (ppm) 4.61 (2H, s, -CH_2_), 7.11-7.17 (2H, m, 2-F-benzyl H_3_ & H_4_), 7.34-7.51 (3H, m, H_8_ & 2-F-benzyl H_5_ & H_6_), 7.60 (1H, t, *J*=7.5 Hz, H_6_), 7.74 (1H, t, *J*= 7.2 Hz, H_7_), 7.83 (2H, d, *J*= 8.5 Hz, phenyl H_2_ & H_6_), 8.14-8.16 (3H, m, H_5_ & phenyl H_3_ & H_5_), 16.31 (1H, s, -OH); LC-MS (ESI) *m/z*: 475.0 (M+1, 100). Anal. Calcd. for C_24_H_15_FN_4_O_4_S; C, 60.76; H, 3.19; N, 11.81. Found: C, 60.90; H, 3.03; N, 11.97.

*3-((4-(5-(4-Fluorobenzylthio)-1, 3, 4-oxadiazol-2-yl) phenyl)diazenyl)-4-hydroxy-2H-chromen-2-one (* ***11h*** *)*

Yield 64%; Orange powder; mp 246.6-251.6 °C; IR (KBr disk): υ (cm^-1^) 1763 (C=O); ^1^HNMR (CDCl_3_, 500 MHz): δ (ppm) 4.56 (2H, s, -CH_2_), 7.07 (2H, t, *J*= 8.5 Hz, 4-F-benzyl H_3_ & H_5_), 7.35-7.41 (2H, m, H_6_ & H_8_), 7.49-7.51 (2H, dd, *J*= 8.2 Hz, 5, 4-F-benzyl H_2_ & H_6_), 7.75 (1H, t, *J*= 7.5 Hz, H_7_), 7.83 (2H, d, *J*= 8.9 Hz, phenyl H_2_ & H_6_), 8.14-8.15 (3H, m, H_8_ & phenyl H_3_ & H_5_), 16.31 (1H, s, -OH); LC-MS (ESI) *m/z*: 475.0 (M+1, 100). Anal. Calcd. for C_24_H_15_FN_4_O_4_S; C, 60.76; H, 3.19; N, 11.81. Found: C, 61.01; H, 3.37; N, 11.69.

**Table 1:** Biological assay and docking results of compounds **11a-h**

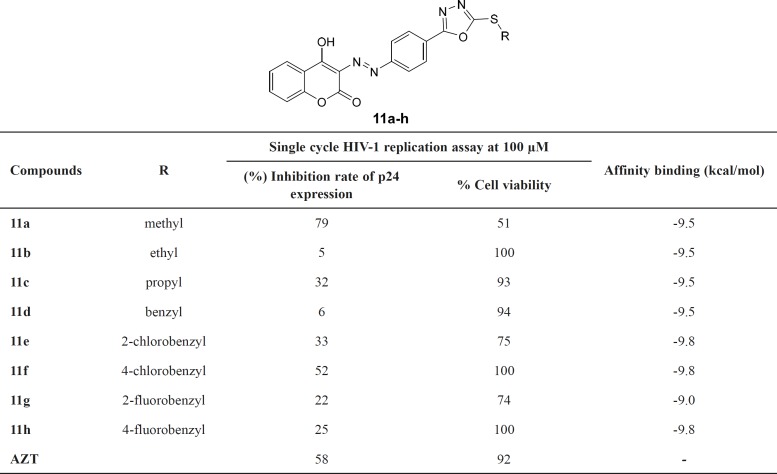

**Table 2:** *In silico *molecular properties calculations of the synthesize compounds **11a-h**.

**No.**	**Log P**	**MW**	**HBA**	**HBD**	**Violations**	**nROTB**	**TPSA**	**ABS%**	**Volume**	**Log S**
**11a**	3.32	380.38	8	1	0	4	114.09	69.64	306.47	-6.300
**11b**	3.82	394.41	8	1	0	5	114.09	69.64	323.27	-6.532
**11c**	4.27	408.44	8	1	0	6	114.09	69.64	340.07	-6.802
**11d**	4.81	456.48	8	1	0	6	114.09	69.64	378.12	-7.881
**11e**	5.41	490.93	8	1	1	6	114.09	69.64	391.65	-8.617
**11f**	5.41	490.93	8	1	1	6	114.09	69.64	391.65	-8.617
**11g**	4.91	474.47	8	1	0	6	114.09	69.64	383.05	-8.195
**11h**	4.91	474.47	8	1	0	6	114.09	69.64	383.05	-8.195
**RAL**	-0.69	444.42	11	3	0	6	152.25	56.47	375.58	-1.978
**ELV**	3.34	447.89	6	2	0	7	88.77	78.37	383.21	-5.655
**DTG**	0.12	419.38	8	2	0	3	100.87	74.20	345.56	-2.579

TPSA: Polar surface area (Å2), %ABS: Absorption percentage, Vol: Volume (Å3), HBA: Number of hydrogen bond acceptor, HBD: Number of hydrogen bond donor, Violation: Number of violation from Lipinski’s rule of five., Log P: Calculated lipophilicity., Log S: Solubility parameter, RAL= raltegravir, ELV= elvitegravir, DTG= dolutegravir.

**Figure 3:**
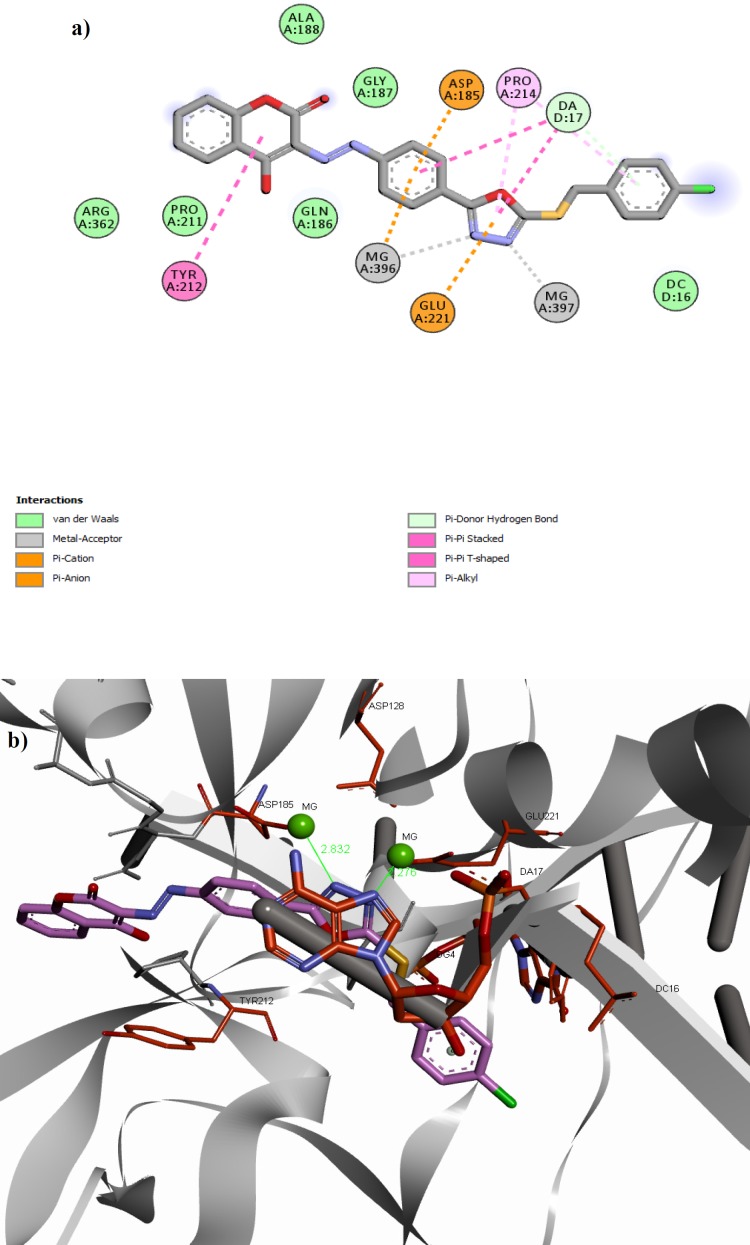
(a) 2D alignment of best docked conformer of compound **11f **and (b) 3D alignment of best docked conformer of compound**11f **(shown in pink) in the PFV IN active site

**Figure 4:**
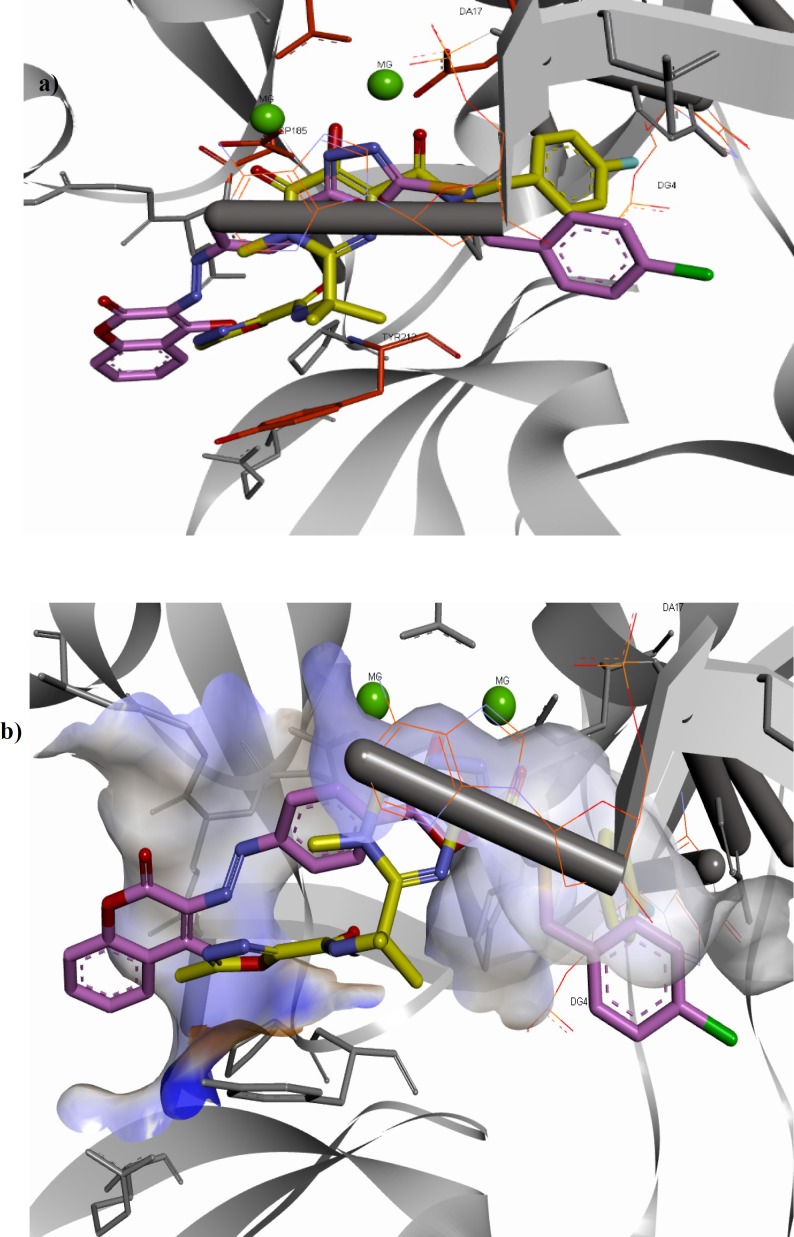
(a) Superimposition of compound **11f **(shown in pink) on raltegravir (shown in yellow) in the PFV IN active site. (b) Interaction of compound **11f **(shown in pink) and raltegravir (shown in yellow) with the surface of PFV IN active site designated with aromatic property

*Molecular modeling studies*

Molecular modeling was performed using the Autodock Vina (28). According to the literature, X-ray crystallographic structure of prototype foamy virus (PFV) IN (PDB: 3OYA) in complex with DNA, two Mg^2+^ cations and raltegravir has many similarities with secondary structure of HIV-IN (RMSD 1.04 Å). Thus, it could be considered as a good model for the development of INSTIs (29). The protein and ligands were prepared in Autodock tools 1.5.6 from MGL Tools package (30). The co-crystallized ligand and water molecules were extracted, Kollman charges were added, nonpolar hydrogens were merged and AutoDock4 atom type assigned to the protein structure. The ligand was created and minimized using HyperChem 8.0 (31). The active site was defined as a Grid box around the crystallographic ligand raltegravir in 20×20×20 dimensions. All molecules were docked in the active site and the bioactive conformations were generated using Autodock Vina.

*In vitro anti-HIV and cytotoxicity assays*

Anti-HIV-1 activity of synthesized compounds was evaluated in single cycle replication assay which was evaluated in our laboratory and reported previously (32-35). In this assay, the single-cycle replicable HIV NL4-3 virions (200 ng p24) were inoculated concurrently with compounds in different concentrations to the Hela cells. The inhibition rate (%) of p24 expression was measured by capture ELISA (Biomerieux, France) 72 hours after inoculation. Percentage inhibition of p24 expression in treated culture was calculated as inhibition rate of p24 (%). The XTT (sodium3-[1(phenylaminocarbonyl)-3,4-tetrazolium]-bis(4-methoxy-6-nitro)benzene sulfonic acid) proliferation assay was conducted to evaluate the cellular toxicity according to kit instructions (36, 37). Cytotoxicity test was performed exactly after the p24 assay. 

## Results and Discussion

*Biological evaluation*

Compounds **11a-h** were assayed for anti-HIV-1 activity in single cycle replication method which reflects only one round of infection. Anti-HIV activity of compounds was measured as inhibition rate (%) of p24 expression in Hela cells cultures. Azidothymidine (AZT) was the positive control in the test. The cytotoxic properties were measured in the same cells by XTT proliferation assay and reported as percentage of cell viability. Results are summarized in Table 1. According to our results, all compounds indicated anti-HIV activity in the range of 5-79% at 100 μM concentration. AZT as positive control exhibited inhibition rate of 58% in our assay. Generally, all the tested compounds proved no considerable cytotoxic properties (cell viability > 74%) except compound **11a** with 51% cell viability. Hence, it can be concluded that the anti-HIV-1 activity of the compounds was not a result of their cytotoxic effects. As it was expected, compounds containing halobenzyl substitutions exhibited better anti-HIV activity as compared to alkyl substitutions. Although compound **11a **containing methyl substitution displayed inhibition rate of 79%, its cell viability was 51% which indicated that its anti-HIV-1 activity might be derived from cytotoxic property. Among alkyl substitutions, propyl (compound **11c**) was reasonably well tolerated, giving similar results to the 2-chlorobenzyl substitution. Introduction of an unsubstituted benzyl group (compound **11d**) was not well tolerated, resulting in reduction in anti-HIV-1 activity (inhibition rate = 6%). Introduction of fluorine atom in position 2 and 4 of benzyl ring (compound **11g** and **11h**, respectively) gave the similar inhibition rates (22 and 25%, respectively). However, compound **11g** with fluorine atom in position 2 showed less %cell viability. The best anti-HIV-1 activity was observed with compounds containing chlorobenzyl substitution (compounds **11e** and **11f**). The shift of the chlorine atom from position 4 (compound **11f**) to position 2 (compound **11e**) was associated with decrease in anti-HIV activity (inhibition rate from 52 to 33%) and increase in cytotoxicity (cell viability from 100 to 75%). It can be concluded that 4-chlorobenzyl moiety at C-5 position of 1,3,4-oxadiazol ring was optimal in terms of anti-HIV activity and cell viability.

*Molecular Modeling and Docking Study*

A molecular docking study was conducted in order to evaluate synthesized compounds interactions with the integrase active site. To validate the docking study, the co-crystalized ligand, raltegravir was redocked under the same condition and superimposed on co-crystalized ligand pose (RMSD = 0.001), and displayed high affinity binding (-12.8 kcal/mol).

Docking results revealed that all the docked compounds accommodated well in the active site with affinity binding energy range from -9.0 to -9.8 kcal/mol (Table 1). Compound **11f** showed high binding affinity (-9.8 kcal/mol). The 3D and 2D alignment of compound **11f** in the active site was shown in Figure 3. The nitrogen atoms of 1,3,4-oxadiazole ring have been located in the 2.28 and 2.83 Å distance from Mg^2+^ essential cofactors and was involved in metal acceptor interaction with the Mg^2+ ^ions. Phenyl and 1,3,4-oxadiazole motifs of the structure have been interacted via π-stacking bonding with deoxy adenosine (DA-17) presenting in the active site and could be expected to displace it. In addition, the 4-hydroxycoumarin motif was able to participate in π-stacking bonding with the Tyr212 residue.

Orientation of compound **11f** and co-crystalized raltegravir in the active site shown in Figure 4 clearly emphasized that both compounds occupy the same place and are engaged in similar interactions with active site residues. Nitrogen atoms of 1,3,4-oxadiazole ring of compound **11f** and chelating motif of raltegravir are nearly superimposable. 4-Chlorobenzyl group of compound **11f** and 4-florobenzyl group of raltegravir have been similarly oriented towards the hydrophobic pocket. 4-Hydroxycoumarin motif of compound **11f** and oxadiazole ring of raltegravir occupy the same area as well.

Collectively, the docking results of tested compounds were in a good agreement with IN inhibitors, suggesting that their anti-HIV activity may be due to IN inhibition. 

*In silico evaluation of drug-likeness*

Oral bioavailability is considered as an important factor in design of new molecules as therapeutic agents. Thus, in drug design it is valuable to gain sufficient information about the molecular properties that limit oral bioavailability. There are some analyses that have correlated physical properties of molecules with successful drug development. Among them, Lipinski′s rule of 5 (38) and Veber′s criteria (39) are the common principles utilized to evaluate the drug-like properties of a compound. In this study, Molinspiration online property calculation toolkit (40) was used to calculate the Lipinski′s molecular properties and the number of rotatable bonds (nROTB), together with the topographical polar surface area (TPSA; a sum of polar atoms′ surfaces: a descriptor for drug absorption, penetrability and bioavailability), the percentage of absorption (ABS%) calculated as (ABS% =109 - 0.345 × TPSA) (41) and the molecular volume (a determinant of the transport characteristics). Lipinski parameters and Veber′s criteria of three FDA-approved INSTIs, raltegravir (RAL), elvitegravir (ELV), and dolutegravir (DTG) were calculated in a same way for comparison. 

All compounds showed log*P* values ranged from 3.32-5.41, MW ranging from 380.38-490.93, HBA value of 8, HBD value of 1, nROTB values ranging from 3-7 (<10), TPSA values lower than 140 Å^2^ (114.09 Å^2^) which all suggesting that the evaluated compounds have acceptable flexibility and are expected to possess good permeability and oral bioavailability (39). According to these calculations, all the compounds displayed the percentage of absorption (ABS%) about 70 which is similar to ABS% of RAL, ELV, and DTG. Additionally, aqueous solubility (Log S) of the tested compounds was determined using OSIRIS Property Explorer (Data Warrior) software (42). As can be seen from Table 2, all the compounds exhibited weak to moderate aqueous solubility with Log S values ranging between -6.300 and -8.617 mol/L which are lower than solubility of RAL, ELV, and DTG.

## Conclusions

The principal objective of this study was to investigate a new chemical scaffold with anti-HIV activity. A novel structure was constructed by hybridization of fragments from well-known anti-HIV-1 lead compounds. Key to the design was the introduction of a 1,3,4-oxadiazole ring as a chelating motif associated with a halobenzyl moiety to mimic pharmacophore of INSTIs. SAR analysis revealed that compound **11f **containing 4-chlorobenzyl ring exhibited best anti-HIV-1 activity among synthesized compounds with inhibition rate of 52% near inhibition rate of AZT (58%). Docking studies showed that designed compounds bind to IN with a mode similar to co-crystalized raltegravir. Moreover, *in silico* computation of ADME properties of designed compounds showed non-violations of Lipinski′s rule of 5 and Veber′s criteria, suggesting that the designed compounds possess favorable physicochemical properties near to those of FDA-approved INSTIs, RAL, ELV, and DTG. However, the designed compounds displayed low aqueous solubility as compared to FDA-approved INSTIs which may be the main reason of their moderate anti-HIV-1 activity. So, these compounds could undergo further structural modifications to improve their anti-HIV-1 activity.
